# Supplementary material for: The impact of the three-level digital divide on the mental health of rural residents: A study from China
Source: PLoS One. 2026 Feb 23;21(2):e0341264. doi: 10.1371/journal.pone.0341264 (PMC12928597; doi:10.1371/journal.pone.0341264)
Supplement: Attachment — Submitted filename: Respond to the reviewers.docx [file pone.0341264.s004.docx]

Respond to the reviewers' comments

Manuscript number :PONE-D-25-20224

Title:The Impact of the Three-Level Digital Divide on the Mental Health of Rural Residents: A Study from China

Dear Editors and Reviewers,

We are most grateful for the opportunity to revise our manuscript entitled "Three-Level Digital Divide on the Mental Health of Rural Residents: A Study from China" and sincerely thank the reviewers for their incisive and constructive comments. Your suggestions have provided invaluable guidance for improving our study and sharpening its scholarly contribution. In the following document we offer a point-by-point response to each reviewer remark; all textual revisions are highlighted in red to distinguish them from the original wording. Reviewers' comments appear in italics, while our modifications and clarifications are presented in red font. We have endeavoured to address every issue raised and trust that the revised version now meets the journal's publication standards.

***editors#***

***comment1：Please ensure that your manuscript meets PLOS ONE's style requirements, including those for file naming.***

Response:Thank you for your guidance. We have meticulously reformatted the entire manuscript in strict accordance with the PLOS ONE style template, including double-spaced 12-point Times New Roman text, Vancouver referencing, and the required title-page layout.

***Comment2：Please ensure that you have an ORCID iD and that it is validated in Editorial Manager.***

Response:Thank you for this reminder. In the revised submission we have added the ORCID iD of the corresponding author, thereby ensuring full compliance with PLOS ONE’s requirements.

***Comment3：Please ensure that you refer to Figure 1 in your text as, if accepted, production will need this reference to link the reader to the figure.***

Response:Thank you for this guidance. In the present revision we have embedded all Figures within the main text so that readers can follow the research logic without interruption.

***Comment4：We are unable to open your Supporting Information file [data1.dta, data2.dta, do.do]. Please kindly revise as necessary and re-upload.***

Response:Thank you for this instruction. In response to the reviewers’ request, we have updated the data files and re-uploaded them. The files “data1.dta” and “data2.dta” contain the raw data for the three-level digital divide, while “data.do” is the full replication code. All three files have been verified to run correctly in Stata and are provided as supporting materials for the reviewers’ inspection.

***Comment5：If the reviewer comments include a recommendation to cite specific previously published works, please review and evaluate these publications to determine whether they are relevant and should be cited. There is no requirement to cite these works unless the editor has indicated otherwise.***

***Comment6：Please review your reference list to ensure that it is complete and correct. If you have cited papers that have been retracted, please include the rationale for doing so in the manuscript text, or remove these references and replace them with relevant current references. Any changes to the reference list should be mentioned in the rebuttal letter that accompanies your revised manuscript. If you need to cite a retracted article, indicate the article’s retracted status in the References list and also include a citation and full reference for the retraction notice.***

Response: Thank you for this guidance. In the present revision we have carefully evaluated every citation for accuracy and relevance, and have updated the reference list accordingly to better reflect the current state of research. The specific adjustments are detailed below.

The deleted literature includes:

1.Huang, C. (2020). The digital divide among the elderly: status, challenges, and strategies. People's Tribune, 2020(12), 126–128.，

2. Li, Z., & Jia, C. (2021). Influence of Internet use on the mental health of middle-aged and older adults: heterogeneity and mechanism test. Jiangsu Social Sciences, 2021(2), 72–79.

3.Chen, L., Jin, S., & Guo, H. (2022). The impact of Internet use on elderly health in rural and urban areas: empirical evidence based on 2018 CHARLS data. Science Research on Aging, 2022(10), 15–28.

4. Sy Atezaz Saeed, &amp;nbsp;Ross M. M. (2021). Disparities in Health Care and the Digital Divide. ACS Applied Materials &amp; Interfaces, 61.

5. Liu, J., & Guo, C. (2021). The impact of mobile Internet application (APP) use on the physical and mental health of the elderly: focusing on WeChat, WeChat moments and mobile payments. Population and Development, 27(2), 117–128.

Updated literature includes:

1. Jun Guo, &amp;nbsp;Wenhao L. (2022). The relationship between the mental health status and social support of the lonely elderly with government participation in the Internet context. Frontiers in Public Health, 10, 1013069.

2. Yifan Yang, &nbsp;Xinying H. (2025). The Multi-Level Influencing Factors of Internet Use Among the Elderly Population and Its Association with Mental Health Promotion: Empirical Research Based on Mixed Cross-Sectional Data. Healthcare, 13 15.

3. Shishuai Fan, &amp;nbsp;Yifan Y. (2022). How Does Internet Use Improve Mental Health among Middle-Aged and Elderly People in Rural Areas in China? A Quasi-Natural Experiment Based on the China Health and Retirement Longitudinal Study (CHARLS). International Journal of Environmental Research and Public Health, 19 20.

4.Jong-Yi Wang, &nbsp;Janice P., &nbsp;Kevin Bennett. (2011). Subdividing the digital divide: differences in internet access and use among rural residents with medical limitations. Journal of Medical Internet Research, 13 1, e25.

5.H. Lee. (2020). Rural and Non-Rural Digital Divide Persists in Older Adults: Internet Access, Usage, and Perception. Innovation in Aging, 4 1, 412–413.

6.Hongwang Guo, &amp;nbsp;Ziming L., &amp;nbsp;Shuyi Feng. (2022). The temperature of internet: Internet use and depression of the elderly in China. Frontiers in Public Health, 10, 1076007.

7. Hu, P., Gong, B., &amp; Fan, W. (2017). Influencing factors on mental health of the elderly under Network Environment. 0.

8.Tang, Y., Li, Q., & Wu, Y. (2024). The impact of the digital divide on rural older People’s mental quality of life: A conditional process analysis. Heliyon, 10(17), e37109.

9.Zhang, S., Wang, Y., & Wei, Y. (2022). Follow or not? Descriptive norms and public health compliance: Mediating role of risk perception and moderating effect of behavioral visibility. Frontiers in Psychology, 13.

10.Naveed Sattar, &nbsp;David P. (2017). Reverse Causality in Cardiovascular Epidemiological Research: More Common Than Imagined? Circulation, 135 24, 2369–2372.

11.Christian Bjerregård Øland. (2024). Reverse causation bias: A simulation study comparing first- and second-line treatments with an overlap of symptoms between treatment indication and studied outcome. PLoS ONE, 19 7, e0304145.

***reviewer1#***

***comment1：In the introduction, the manuscript defines its sample as "rural residents" but heavily emphasizes "farmers”. If "rural residents" is the broader focus, the introduction needs to acknowledge the diversity within this group and include literature review about the remaining group of the population.***

Response:Thank you for highlighting this issue. We now recognise that "rural residents" is a broader category than "farmers". To avoid ambiguity, we have revised the Introduction to clearly distinguish between the two concepts and have added literature covering non-farmer rural populations (e.g., rural self-employed, left-behind elderly, village teachers) to ensure that the study background aligns with our sample definition.

The specific revisions can be found in the following two sections.

On the fourth page：In the context of the digital divide, rural residents constitute a distinct and deeply affected group. Although often collectively referred to as farmers, this population in fact displays significant occupational heterogeneity, encompassing not only those engaged in agricultural production but also local non-agricultural workers, return-migrant entrepreneurs, retirees, and other long-term inhabitants of rural China—rather than being restricted to farmers in the traditional sense. Consequently, the present study is situated within this demographically diverse rural population rather than being limited to individuals whose primary activity is farming.

And on the tenth page：Moreover, although existing research has predominantly focused on agricultural populations in rural areas, mounting evidence indicates that non-agricultural rural residents—such as return-migrant entrepreneurs, local non-farm workers, and retirees—also face a twofold disadvantage stemming from both digital access divides and insufficient digital-use capabilities. For instance, Wang found that rural residents’ access to health care and mental-health treatment is constrained by limitations in internet access and use[27]. Lee further observed that among older rural residents the effects of internet access, use, and perceived utility are more severe than among their non-rural counterparts and vary across occupations. These studies suggest that the impact of the digital divide may exhibit intra-rural heterogeneity contingent upon occupational status and social identity[28].

***Comment2：Within the methods section, an explicit statement detailing the ethical approval for the original CFPS data collection and how participant consent was obtained must be included, even for secondary data use.***

Response:Thank you for this reminder. We have added a statement in the Methods section clarifying that the original CFPS data collection was approved by the Peking University Institute of Social Science Survey (ISSS) in accordance with the Chinese Ethical Review Measures, and informed consent was obtained from all respondents. As our study uses only anonymised secondary data, no additional ethical review was required; the approval number and consent procedures are now detailed in the revised manuscript.

The specific revision can be found on page 19 of the revised manuscript.

The China Family Panel Studies (CFPS) has been approved by the Institutional Review Board of Peking University (approval No. IRB00001052-14010). All respondents provided written informed consent prior to data collection. The CFPS data are de-identified and publicly released by the Institute of Social Science Survey at Peking University. As this study uses only anonymized secondary data, no additional ethical review is required.

***Comment3：Also in the methods, since new composite variables (e.g., mental health, digital use, digital utility) were created using PCA, psychometric evidence for these in the sample must be provided.***

Response:Thank you for this helpful reminder. We have now added a new subsection, “Psychometric properties of composite variables,” to the Methods. There we systematically report the total variance explained by the retained PCA components, the range of factor loadings, Cronbach’s α, and the inter-item correlation matrix for each index. On the basis of these updated, higher-explanation composites we re-estimated all models that use these variables, thereby ensuring the reliability and internal consistency of the subsequent estimates.

The specific revision can be found on page 20 of the revised manuscript.

3.2 Psychometric Properties of Composite Variables

Principal-component analysis (PCA) was used to construct composite indices for mental health, digital use, and digital utility. The mental-health scale was derived from eight items; four principal components were retained, explaining 74.3 % of the total variance (first component = 39.9 %). Item loadings ranged from 0.24 to 0.43. Cronbach’s α = 0.80, indicating good internal consistency; mean inter-item correlation = 0.30 (range 0.17–0.52). The composite score was approximately normal (M = 0.00, SD = 1.00). The digital-use-divide index was derived from four items; three principal components were retained, explaining 86.2 % of the variance (first component = 36.5 %). Loadings ranged from 0.15 to 0.69. Cronbach’s α = 0.35, denoting modest reliability; mean inter-item correlation = 0.06 (range 0.04–0.11). The composite was roughly normal (M = 0.00, SD = 0.66). The digital-utility-divide index was derived from five items; the retained components explained 79.2 % of the variance (first component = 36.5 %). Loadings ranged from 0.26 to 0.52. Cronbach’s α = 0.73, indicating good internal consistency; mean inter-item correlation = 0.36 (range 0.16–0.50). The composite score was approximately normal (M = 0.00, SD = 0.98). Table 2 presents the detailed component loadings for each item. For mental health, “felt down” and “everything an effort” loaded positively on the first component, contributing to the overall score. For digital use, “online gaming” and “online learning” showed salient loadings on the first component. For digital utility, “importance for work” and “importance for entertainment” loaded on the first component. These patterns support the factorial validity of the indices, indicating that the items adequately capture the intended constructs.

Table 1:Psychometric properties of PCA-based composites

| Construct | Items | Retained PCs | Cumulative variance (%) | Cronbach α | Loading range | Item correlations | Composite M±SD |
| --- | --- | --- | --- | --- | --- | --- | --- |
| Mental Health Levels | 8 | 4 | 74.3 | 0.78 | 0.24-0.43 | 0.30(0.17–0.52) | 0.00 ± 1.00 |
| Digital Use Divide | 4 | 3 | 86.2 | 0.35 | 0.15–0.69 | 0.06 (0.04–0.11) | 0.00 ± 0.66 |
| Digital Utility Divide | 5 | 3 | 79.2 | 0.73 | 0.26–0.52 | 0.36 (0.16–0.50) | 0.00 ± 0.98 |

Table 2:Loadings for PCA-based Composites

| Item | Construct | Comp1 | Comp2 | Comp3 | Comp4 | Unexplained |
| --- | --- | --- | --- | --- | --- | --- |
| How often have you felt pleased in the past week | Mental health | 0.237 | 0.668 | 0.122 | 0.012 | 0.219 |
| How often have you felt happy in life in the past week | Mental health | 0.258 | 0.646 | 0.027 | 0.008 | 0.235 |
| How often have you felt down in the past week? | Mental health | 0.381 | -0.154 | 0.323 | -0.553 | 0.211 |
| How often have you felt that everything is an effort in the past week | Mental health | 0.381 | -0.209 | 0.400 | -0.305 | 0.289 |
| How often have you had trouble falling asleep in the past week | Mental health | 0.329 | -0.185 | 0.651 | 0.625 | 0.020 |
| How often have you felt lonely in the past week | Mental health | 0.390 | -0.125 | -0.311 | 0.195 | 0.411 |
| How often have you felt sad in the past week | Mental health | 0.426 | -0.089 | -0.355 | 0.013 | 0.311 |
| How often have you felt sad in the past week | Mental health | 0.382 | -0.103 | -0.516 | 0.068 | 0.306 |
| Do you play online games daily | Digital use | 0.149 | 0.694 | -0.701 | - | 0.003 |
| Do you use the internet for learning daily | Digital use | 0.695 | -0.122 | -0.048 | - | 0.277 |
| Do you use short videos daily | Digital use | 0.688 | -0.175 | 0.047 | - | 0.273 |
| Do you shop online daily | Digital use | 0.148 | 0.688 | 0.710 | - | 0.000 |
| How important is internet learning for work | Digital utility | 0.485 | -0.236 | 0.055 | - | 0.211 |
| How important is the internet for entertainment | Digital utility | 0.452 | -0.128 | 0.782 | - | 0.096 |
| How important is the internet for keeping in touch with family and friends | Digital utility | 0.264 | 0.956 | 0.055 | - | 0.012 |
| How important is internet learning for work | Digital utility | 0.515 | -0.096 | -0.241 | - | 0.315 |
| How important is the internet for daily life | Digital utility | 0.476 | -0.066 | -0.570 | - | 0.239 |

***Comment4：Regarding variable detailing in the methods, when explaining variables in the text, it is not necessary to list every single item if the full details are already provided in a table. A concise explanation with a few key examples is sufficient in the paragraph.***

Response:Thank you for this suggestion. Following the principle of “key examples only in the text, full details in tables,” we have streamlined the variable descriptions: the main text now presents the core construct and two-to-three illustrative items, while exhaustive item wordings and loadings remain in the corresponding tables. This keeps the narrative concise without loss of information or replicability.

The specific revision can be found on page 23 of the revised manuscript.

As previously noted, this study follows Li’s multidimensional approach to assessing respondents’ mental-health status, drawing on the relevant battery of items contained in the CFPS2020 and CFPS2022 questionnaires[53]. The items ask how often during the past week respondents experienced specific feelings such as “felt down,” “felt lonely,” “felt sad,” as well as positive affects “felt pleased” and “felt happy.” Answers are recorded on a four-point scale ranging from “most of the time (5–7 days)” to “almost never (<1 day),” coded 1–4. A composite mental-health score was created by principal-component analysis (PCA), with higher values denoting better mental health. To ensure consistent polarity, the two positive-affect items (“felt pleased” and “felt happy”) were reverse-coded so that higher scores reflect more frequent positive affect.

The specific revision can be found on page 24 of the revised manuscript.

For the Digital Use Divide,the indicator is constructed from respondents’ frequency and purpose of internet use. Among those not affected by the digital access divide, the questionnaire records whether they engage in four online activities (online gaming, online learning, short-video viewing, and online shopping). A “yes” response is coded 0 (no use divide), whereas “no” is coded 1 (divide present). These four items were submitted to principal-component analysis (PCA) to create the “digital use divide” variable; higher values signify a more pronounced digital use divide.

The digital utility divide is determined by the perceived importance of the internet. For respondents unaffected by the digital access divide, the questionnaire assesses the importance of the internet in five domains: learning, work, staying in touch with family and friends, entertainment, and daily life. Each item is rated on a five-point scale from “very important” to “very unimportant,” coded 1–5. These five items were combined via principal-component analysis (PCA) into a single “digital utility divide” variable; higher values indicate lower perceived importance of the internet and thus a more severe digital utility divide.

***Comment5：Finally, the paper lacks a dedicated "Discussion" section. A standalone discussion is essential for synthesizing findings, comparing them with existing literature, and exploring theoretical and policy implications in depth.***

Response:Thank you for this valuable suggestion. We have added a stand-alone “6 Discussion” section that synthesizes our findings against the domestic and international literature of the past three years, clarifies the theoretical contributions and policy implications, and critically examines potential reverse-causality pathways. We also outline directions for future experimental designs, thereby meeting the requirement for an in-depth discussion.

The specific revisions can be found on page 50 of the revised manuscript.

6 Discussion The continuous advancement and popularization of internet technology have transformed the digital divide from a primary "digital access divide" into second-level "digital use divide" and third-level "digital utility divide." Using data from CFPS2020 and CFPS2022, this study deeply explores the impact of the digital divide on the mental health of rural Chinese residents. Existing research on this topic is relatively limited, with most studies focusing on the effects of the digital access and use divides, while the impact of the digital utility divide on rural residents’ mental health remains under-researched.

The findings reveal that when rural residents lack internet access, exhibit low internet usage frequency, or perceive low importance of the internet, their mental health declines. Sub-dimension analysis shows that the absence or improper use of online learning skills, online shopping skills, and short video skills are associated with reduced mental health. Additionally, the negative impact of internet use on maintaining contact with family and friends also adversely affects rural residents’ mental health. Furthermore, the influence of the digital divide on mental health is moderated by factors such as age, gender, education level, and regional location. Specifically:The digital access divide impairs mental health by reducing residents’ Self-Assessed Sense of Fairness. The digital use divide lowers mental health through decreasing Self-Assessed Social Class Perception. The digital utility divide affects mental health by reducing both Self-Assessed Social Class Perception and Self-Assessed Economic Status.

Relative to the existing literature, the present study makes two principal contributions. First, we extend the conventional two-tier conceptualisation of the digital divide (access versus use) to a three-tier framework by incorporating the digital utility divide. Prior work has overwhelmingly operationalised the divide as a binary of “access–use”, thereby neglecting the deeper implications of perceived internet utility. Our findings not only furnish robust evidence for the existence of the utility divide, but also dissect its nuanced psychological consequences for rural residents. This theoretical extension furnishes an analytical template for future scholarship. Second, we offer a focused examination of rural populations in a developing-country context, thereby redressing the paucity of mental-health research on this demographic. Rather than restricting attention to canonical “farmers”, we broaden the analytical lens to encompass all agricultural and non-agricultural workers resident in rural areas—an innovation that carries direct implications for place-based policy design. Additionally, by disaggregating the three divides and tracing their differential pathways—via self-assessed social class, self-assessed economic status, and perceived fairness—we provide a level of mechanistic detail that is seldom available in extant studies and that is indispensable for targeted intervention. Notwithstanding these strengths, full resolution of reverse causality remains elusive. Rural Chinese residents confront objectively constrained social channels and cognitive repertoires relative to their urban counterparts. Following Sattar and Øland , two feedback loops are plausible. First, depressive symptomatology may precipitate social withdrawal, eroding the perceived need to go online; low self-efficacy leads individuals to forgo the internet as a means of social or informational expansion, manifesting as access and use divides[64]. Second, depression can impair technology-learning capacity, causing already-connected individuals to abandon skill acquisition and to devalue internet utility—an endogenous process that surfaces as a utility divide. Future work should employ randomised or cross-over designs to neutralise these reciprocal influences[65]. Nevertheless, the battery of endogeneity tests deployed here—IV estimation, Hausman specification tests, and partial-R² metrics—together with an extensive suite of robustness checks, sustains the credibility and policy relevance of the core findings.

***reviewe2#***

***Comment1：Provide more detail on the PCA used to derive the mental health, digital use, and digital utility indices, including loadings, explained variance, and reliability tests.***

Response:Thank you for this valuable suggestion. We have now added complete PCA metrics (loadings, explained variance, and reliability) to the Methods section; see Section 3.2 and Table 2. Concurrently, we re-estimated all empirical models and tests involving the PCA-based composites using the new, higher-explanation versions to ensure the robustness and reliability of our findings.

The specific revisions can be found on page 20 of the revised manuscript.

3.2 Psychometric Properties of Composite Variables

Principal-component analysis (PCA) was used to construct composite indices for mental health, digital use, and digital utility. The mental-health scale was derived from eight items; four principal components were retained, explaining 74.3 % of the total variance (first component = 39.9 %). Item loadings ranged from 0.24 to 0.43. Cronbach’s α = 0.80, indicating good internal consistency; mean inter-item correlation = 0.30 (range 0.17–0.52). The composite score was approximately normal (M = 0.00, SD = 1.00). The digital-use-divide index was derived from four items; three principal components were retained, explaining 86.2 % of the variance (first component = 36.5 %). Loadings ranged from 0.15 to 0.69. Cronbach’s α = 0.35, denoting modest reliability; mean inter-item correlation = 0.06 (range 0.04–0.11). The composite was roughly normal (M = 0.00, SD = 0.66). The digital-utility-divide index was derived from five items; the retained components explained 79.2 % of the variance (first component = 36.5 %). Loadings ranged from 0.26 to 0.52. Cronbach’s α = 0.73, indicating good internal consistency; mean inter-item correlation = 0.36 (range 0.16–0.50). The composite score was approximately normal (M = 0.00, SD = 0.98). Table 2 presents the detailed component loadings for each item. For mental health, “felt down” and “everything an effort” loaded positively on the first component, contributing to the overall score. For digital use, “online gaming” and “online learning” showed salient loadings on the first component. For digital utility, “importance for work” and “importance for entertainment” loaded on the first component. These patterns support the factorial validity of the indices, indicating that the items adequately capture the intended constructs.

Table 1:Psychometric properties of PCA-based composites

| Construct | Items | Retained PCs | Cumulative variance (%) | Cronbach α | Loading range | Item correlations | Composite M±SD |
| --- | --- | --- | --- | --- | --- | --- | --- |
| Mental Health Levels | 8 | 4 | 74.3 | 0.78 | 0.24-0.43 | 0.30(0.17–0.52) | 0.00 ± 1.00 |
| Digital Use Divide | 4 | 3 | 86.2 | 0.35 | 0.15–0.69 | 0.06 (0.04–0.11) | 0.00 ± 0.66 |
| Digital Utility Divide | 5 | 3 | 79.2 | 0.73 | 0.26–0.52 | 0.36 (0.16–0.50) | 0.00 ± 0.98 |

Table 2:Loadings for PCA-based Composites

| Item | Construct | Comp1 | Comp2 | Comp3 | Comp4 | Unexplained |
| --- | --- | --- | --- | --- | --- | --- |
| How often have you felt pleased in the past week | Mental health | 0.237 | 0.668 | 0.122 | 0.012 | 0.219 |
| How often have you felt happy in life in the past week | Mental health | 0.258 | 0.646 | 0.027 | 0.008 | 0.235 |
| How often have you felt down in the past week? | Mental health | 0.381 | -0.154 | 0.323 | -0.553 | 0.211 |
| How often have you felt that everything is an effort in the past week | Mental health | 0.381 | -0.209 | 0.400 | -0.305 | 0.289 |
| How often have you had trouble falling asleep in the past week | Mental health | 0.329 | -0.185 | 0.651 | 0.625 | 0.020 |
| How often have you felt lonely in the past week | Mental health | 0.390 | -0.125 | -0.311 | 0.195 | 0.411 |
| How often have you felt sad in the past week | Mental health | 0.426 | -0.089 | -0.355 | 0.013 | 0.311 |
| How often have you felt sad in the past week | Mental health | 0.382 | -0.103 | -0.516 | 0.068 | 0.306 |
| Do you play online games daily | Digital use | 0.149 | 0.694 | -0.701 | - | 0.003 |
| Do you use the internet for learning daily | Digital use | 0.695 | -0.122 | -0.048 | - | 0.277 |
| Do you use short videos daily | Digital use | 0.688 | -0.175 | 0.047 | - | 0.273 |
| Do you shop online daily | Digital use | 0.148 | 0.688 | 0.710 | - | 0.000 |
| How important is internet learning for work | Digital utility | 0.485 | -0.236 | 0.055 | - | 0.211 |
| How important is the internet for entertainment | Digital utility | 0.452 | -0.128 | 0.782 | - | 0.096 |
| How important is the internet for keeping in touch with family and friends | Digital utility | 0.264 | 0.956 | 0.055 | - | 0.012 |
| How important is internet learning for work | Digital utility | 0.515 | -0.096 | -0.241 | - | 0.315 |
| How important is the internet for daily life | Digital utility | 0.476 | -0.066 | -0.570 | - | 0.239 |

***Comment2：Clarify handling of missing data and whether sample weights were applied.***

Response:Thank you for this reminder. We have now clarified in the "Data and Sample" section that missing values were handled by list-wise deletion: only observations with complete 2020–2022 longitudinal matches and non-missing key variables were retained, yielding 11,114 valid cases. As CFPS does not supply survey weights, our estimates are unweighted. To mitigate potential bias we control for province- and individual-level covariates and conduct multiple robustness checks, with full details reported in the main text and Appendix.

The specific revisions can be found on page 20 of the revised manuscript.

In our study, we carefully handled missing data and considered the use of sample weights to ensure the robustness and representativeness of our analyses. Missing data were addressed through stringent procedures to preserve data integrity. Specifically, we excluded observations that were not successfully matched across the 2020 and 2022 waves of the China Family Panel Studies (CFPS) and dropped cases with missing values on key independent or dependent variables, yielding a final analytic sample of 11,114 valid observations.

Regarding weights, the CFPS data set does not provide sampling weights; consequently, our analyses rely on unweighted data. We have evaluated the representativeness of our sample and consider it to adequately reflect the characteristics of rural Chinese residents, given that CFPS employs a stratified sampling design covering 25 provinces/autonomous regions. To mitigate potential biases arising from the absence of sampling weights and from item non-response, we control for a comprehensive set of individual- and household-level covariates and conduct extensive robustness checks. These measures jointly enhance the reliability and validity of our findings. Moreover, a number of published studies likewise omit weight adjustments and still obtain widely accepted results; in causal-inference settings such as ours, the influence of weighting is typically of secondary importance.

***Comment3：Clarify handling of missing data and whether sample weights were applied.***

Response:Thank you for this helpful suggestion. We have substantially expanded Section 4.3 into a two-part justification: the first paragraph derives the IV from the administrative “universal-service” quota and village-level infrastructure thresholds, while the second invokes peer-threshold theory to defend the exclusion restriction. All accompanying first-stage diagnostics (F > 600, partial-R² > 0.70, Hausman tests) are now reported in the revised text and in Table 7, fully satisfying the requested diagnostics.

The specific revisions can be found on page 37 of the revised manuscript.

The theoretical justification for treating the village-level, same-age-cohort mean of each digital-divide dimension as an exogenous shock rests on the supply-side determinants of rural internet access. Experimental evidence shows that China’s “Universal Service” fibre-to-the-village programme allocated roll-out quotas to administrative villages by administrative decree, generating discontinuous jumps in network availability that are orthogonal to household-level unobservables [59]. Because the “last-mile” investment is negotiated by village committees and subsidised by county governments, the proportion of a specific age group within a village that remains unconnected, uses the internet infrequently, or attributes low utility to it is driven primarily by these exogenous infrastructure thresholds rather than by individual psycho-social traits. The cohort mean therefore captures a community-level supply shock that satisfies the relevance condition and is systematically unrelated to the idiosyncratic component of mental-health outcomes.

Second, social-norms and reference-group theory provide complementary support for the exclusion restriction. Zhang demonstrate that, once network coverage is in place, peer visibility within the village creates a threshold effect: adoption accelerates as soon as the peer-use rate crosses a critical point, independently of individual socioeconomic status[60]. Since beliefs about mental health in rural China are formed through local rather than cross-village comparisons, the village–age-group mean influences psychological well-being only by shaping an individual’s own digital access, intensity of use, and perceived utility. The three instruments thus isolate exogenous community-level variation in the digital access, use, and utility divides, enabling causal identification of each divide’s effect on mental health.

The IV results are reported in Table 7. After instrumenting, the digital access divide continues to exert a negative effect on rural residents’ mental health. In the first stage the F-statistic equals 723.72—far above the conventional threshold of 10—so the weak-instrument test is easily passed. The instrument is positively and significantly related to the access divide at the 1 % level, confirming relevance. The Hausman statistic is 0.02 with a p-value of 1.00, indicating that the access divide can be treated as exogenous. The IV estimate remains negative and significant at the 10 % level, corroborating robustness; the first-stage partial R² is 0.7013, showing that the instrument explains a large share of the variation in the endogenous regressor.

For the digital use divide, the first-stage coefficient is 0.9890 (significant at 1 %), and the F-statistic is 718.68, again rejecting the weak-instrument hypothesis. The second-stage coefficient is negative and significant at 1 %. The Hausman test yields 1.40 (p = 0.9992), so the use divide is also exogenous. The partial R² reaches 0.7425, reaffirming instrument strength.

Turning to the digital utility divide, the first-stage coefficient equals 0.8980 (significant at 1 %), with an F-statistic of 615.12. The IV estimate is −0.0427 and significant at 10 %. The Hausman statistic is 0.29 (p = 1.00), and the partial R² is 0.7534, demonstrating strong explanatory power.

Taken together, the instruments perform well: partial R² values of 0.7013, 0.7425 and 0.7534 imply a high correlation with the respective endogenous variables, while the large first-stage F-statistics and high Hausman p-values jointly establish instrument exogeneity and validity. Thus, the baseline findings are robust to endogeneity concerns: all three layers of the digital divide significantly undermine the mental health of rural Chinese residents.

***Comment4：The ethics section currently states “N/A,” which is not acceptable. Explicitly describe the IRB approvals from CFPS, informed consent procedures, and compliance with data-use agreements.***

Response:Thank you for emphasising the need for a full ethics statement. In the revised manuscript we have replaced the previous “N/A” with the following declaration: “The China Family Panel Studies (CFPS) received ethical approval from the Institutional Review Board of Peking University (approval No. IRB00001052-14010). All participants provided written informed consent prior to data collection. The de-identified CFPS datasets are publicly released by the Institute of Social Science Survey, Peking University. As this study uses only anonymous secondary data, no additional ethical review was required.” This revision explicitly documents the IRB approval, informed-consent procedures, and compliance with the data-use agreement, thereby meeting the journal’s requirements.

The specific revisions can be found on page 19 of the revised manuscript.

The China Family Panel Studies (CFPS) has been approved by the Institutional Review Board of Peking University (approval No. IRB00001052-14010). All respondents provided written informed consent prior to data collection. The CFPS data are de-identified and publicly released by the Institute of Social Science Survey at Peking University. As this study uses only anonymized secondary data, no additional ethical review is required.

***Comment5：Include 95% confidence intervals and standardized coefficients to allow better interpretation of effect sizes.***

Response:Thank you for this helpful suggestion. We have now added 95 % confidence intervals and standardized coefficients to Table 5 to facilitate interpretation of effect sizes. Columns (1)–(3) show that the 95 % CIs for the digital-access divide (−0.1959, −0.0237), the digital-use divide (−0.1886, −0.0982) and the digital-utility divide (−0.1468, −0.0703) lie entirely below zero, underscoring the precision and reliability of the negative effects. Columns (4)–(6) present standardized coefficients, enabling effect-size comparisons. The standardized beta is −0.0537 (p < 0.05) for the digital-access divide, −0.0946 (p < 0.01) for the digital-use divide and −0.1070 (p < 0.01) for the digital-utility divide. These results indicate that while all three layers of the digital divide harm mental health, interventions targeting the digital-utility divide are likely to yield the largest mental-health gains.

The specific revisions can be found on page 32 of the revised manuscript.

To sharpen the estimated impact of each digital-divide layer on rural residents’ mental health, Table 5 reports 95 % confidence intervals and standardized coefficients. Columns (1)–(3) show that the 95 % CIs for the digital access divide (−0.1959, −0.0237), the digital use divide (−0.1886, −0.0982) and the digital utility divide (−0.1468, −0.0703) are entirely below zero, underscoring the precision and reliability of the negative effects. Columns (4)–(6) present standardized coefficients that permit effect-size comparisons across metrics. The standardized beta for the digital access divide is −0.0537 (p < 0.05), indicating a medium effect. The corresponding values for the digital use divide and the digital utility divide are −0.0946 and −0.1070 (both p < 0.01), respectively. Thus, the digital utility divide exerts the largest relative influence, followed by the digital use divide, while the digital access divide shows the smallest standardized impact. These results imply that—although all three layers harm mental health—interventions that heighten rural residents’ perceived value of internet use (i.e., reduce the digital utility divide) are likely to yield the largest mental-health gains. Standardized coefficients therefore offer a clear empirical basis for prioritizing policies that enhance perceived internet utility alongside continued efforts to improve access and usage skills.

### Table 5: Digital Divide's Impact on Rural Residents' Mental Health (with 95% CIs & Std. Coeff.)

| Item | （1） | （2） | （3） | （4） | （5） | （6） |  |  |  |
| --- | --- | --- | --- | --- | --- | --- | --- | --- | --- |
| Digital Access Divide | [-0.1959,-0.0237] |  |  | -0.0537**  (-2.50) |  |  |  |  |  |
| Digital Use Divide |  | [-0.1886, -0.0982] |  |  | -0.0946*** (-6.22) |  |  |  |  |
| Digital Utility Divide |  |  | [-0.1468, -0.0703] |  |  | -0.1070*** (-5.57) |  |  |  |
| Gender | [-0.8866,0.6129] | [-0.3454, 1.2943] | [-0.1825, 1.1367] | -0.1380  (-0.36) | 0.4760  (1.13) | 0.4790 (1.42) |  |  |  |
| edu | [-0.4456,-0.0032] | [-0.4240, 0.0639] | [-0.4228, 0.0560] | -0.2250**  (-1.99) | -0.1810 (-1.45) | -0.1840 (-1.50) |  |  |  |
| marriage | [-0.0261, 0.4833] | [0.0987, 0.6489] | [0.0893, 0.6352] | 0.2300*  (1.76) | 0.3750*** (2.66) | 0.3630*** (2.60) |  |  |  |
| age | [-0.0117, 0.0137] | [0.0987, 0.6489] | [-0.0216, 0.0126] | 0.0010  (0.15) | -0.0027 (-0.30) | -0.004 (-0.52) |  |  |  |
| religion | [-0.1641, 0.1371] | [-0.1755, 0.3723] | [-0.1978, 0.3510] | -0.0136  (-0.18) | 0.0987 (0.70) | 0.0768 (0.55) |  |  |  |
| fml_count | [-0.0007, 0.0393] | [-0.0093, 0.0409] | [-0.0090, 0.0410] | 0.0193*  (1.89) | 0.0159 (1.24) | 0.0161 (1.25) |  |  |  |
| house | [0.0067, 0.2932] | [-0.1782, 0.2305] | [-0.1736, 0.2373] | 0.1510**  (2.05) | 0.0262 (0.25) | 0.0319 (0.30) |  |  |  |
| car | [-0.0258, 0.2326] | [-0.0234, 0.3387] | [-0.0133, 0.3463] | 0.1040 (1.57) | 0.158* (1.71) | 0.167* (1.82) |  |  |  |
| [region](https://fanyi.so.com/?src=onebox#region)1 | Control | | | | | |  |  |  |
| [region](https://fanyi.so.com/?src=onebox#region)2 | Control | | | | | |  | 11114 |  |
| N | 11114 | 6815 | 6815 | 11114 | 6815 | 6815 |  |  |  |
| Notes:  ① *, **, and *** denote significance at the 10%, 5%, and 1% levels, respectively.  ② Columns (1)–(3) report 95 % confidence intervals of the estimated coefficients to assess precision.  ③ Columns (4)–(6) report standardized coefficients to facilitate comparison of effect sizes across variables.  ④ Individual and year fixed effects are controlled for. | | | | | | |  |  |  |

***Comment6：Summarize key findings more concisely in the text to complement complex tables.***

Response:Thank you for your suggestion. We have streamlined the text to more succinctly summarize the key findings, ensuring that the main results are clearly highlighted alongside the detailed tables. This enhances the readability and accessibility of the manuscript, making it more broadly applicable to diverse readerships.

The corresponding revisions are highlighted in red on pages 32, 40, 45 and 46, as well as in all other amended sections of the revised manuscript.

pages 32:

The IV results are reported in Table 7. After instrumenting, the digital access divide continues to exert a negative effect on rural residents’ mental health. In the first stage the F-statistic equals 723.72—far above the conventional threshold of 10—so the weak-instrument test is easily passed. The instrument is positively and significantly related to the access divide at the 1 % level, confirming relevance. The Hausman statistic is 0.02 with a p-value of 1.00, indicating that the access divide can be treated as exogenous. The IV estimate remains negative and significant at the 10 % level, corroborating robustness; the first-stage partial R² is 0.7013, showing that the instrument explains a large share of the variation in the endogenous regressor.For the digital use divide, the first-stage coefficient is 0.9890 (significant at 1 %), and the F-statistic is 718.68, again rejecting the weak-instrument hypothesis. The second-stage coefficient is negative and significant at 1 %. The Hausman test yields 1.40 (p = 0.9992), so the use divide is also exogenous. The partial R² reaches 0.7425, reaffirming instrument strength.Turning to the digital utility divide, the first-stage coefficient equals 0.8980 (significant at 1 %), with an F-statistic of 615.12. The IV estimate is −0.0427 and significant at 10 %. The Hausman statistic is 0.29 (p = 1.00), and the partial R² is 0.7534, demonstrating strong explanatory power.

Taken together, the instruments perform well: partial R² values of 0.7013, 0.7425 and 0.7534 imply a high correlation with the respective endogenous variables, while the large first-stage F-statistics and high Hausman p-values jointly establish instrument exogeneity and validity. Thus, the baseline findings are robust to endogeneity concerns: all three layers of the digital divide significantly undermine the mental health of rural Chinese residents.

Pages40:

4.4 Sub-Dimension Analysis.The disaggregated results in Table 8 show that the frequency with which rural residents play online games has no discernible effect on their mental-health score. By contrast, skills related to online learning, e-shopping, and the use of new-media platforms (short videos) are all positively and significantly associated with better mental health; respondents who report lower proficiency in these domains exhibit markedly poorer psychological well-being. Although online gaming has expanded rapidly and is widely recognised, rural residents—who on average have lower educational attainment and limited digital literacy—often lack the competencies needed to engage meaningfully with games; this plausibly explains the null finding for gaming frequency. The remaining dimensions of the digital-use divide are therefore more salient for rural residents, because they govern the ability to access information safely and conveniently and to improve daily living. Deficiencies in these practical skills translate directly into lower mental-health scores.

Table 9 turns to the sub-dimensions of the digital-utility divide. Perceived importance of the internet for “staying in touch with family and friends” is negatively associated with mental health at the 1 % level: the higher the subjective value placed on online interpersonal contact, the worse the psychological outcome. Utilities attached to work, entertainment, learning and daily life, however, are statistically insignificant. A likely explanation is the socio-economic context of rural China: villages are relatively isolated and economically under-developed, prompting many working-age adults to migrate to cities. When the internet fails to deliver the expected relational benefits—i.e., when the utility divide in the social-connectivity domain is wide—feelings of loneliness intensify and mental health deteriorates. Lower average schooling and lagging local economic development may also attenuate the psychological relevance of internet utilities outside the interpersonal sphere, producing the non-significant results observed for the other domains.

pages 45:

Age Heterogeneity. Drawing on Zhao’s research, the sample was divided into two groups: young respondents (under 45 years old) and middle-aged and elderly respondents (45 years old and above) [61]. The results in Table 10 indicate that, among younger respondents, poorer mental health is driven primarily by the digital access and digital use divides. In sharp contrast, for middle-aged and elderly villagers the digital use divide alone exerts the most pronounced negative effect. This age-pattern likely reflects the life-course realities of rural China: older adults need a broader repertoire of online skills—job search, mobile banking, government-service portals—to secure or retain off-farm employment, so deficiencies in use directly curtail their labour-market opportunities and, in turn, depress psychological well-being. Younger villagers, while more digitally native, still face fundamental barriers to entry (device affordability, village-level fibre availability); once on-line, they demand advanced skills for study, social media and e-commerce. Hence inadequate access and low usage intensity reduce their information-gathering efficiency and generate the observed mental-health penalty.

Gender Heterogeneity. Following Li’s (2025) methodology, all respondents were divided into two groups by gender for in-depth analysis[55]. The results show that men’s mental health is impaired mainly by the digital use and digital utility divides, whereas women are adversely affected across all three layers. Along both the access and use dimensions, the negative mental-health effect is significantly larger for women; along the utility dimension, the effect is larger for men. These patterns plausibly reflect regional disparities in economic development and gendered educational expectations. Women, who on average face lower access to and fewer opportunities for internet use, tend to focus on its benefits and are less aware of potential downsides; hence each additional barrier to connectivity or usage translates into a sharper psychological penalty. Men, enjoying greater access and usage experience, are more attuned to the risks and limitations of online activity, so the perceived (low) utility of the internet exerts a stronger negative influence on their well-being.

Regional Heterogeneity. Following Yang’s research, respondents were divided into two groups by province: eastern region (residing in eastern China) and non-eastern region (central and western China)[8], with results presented in Table 10. Empirical findings reveal marked regional heterogeneity in the relationship between the digital divide and rural residents’ mental health. Specifically, the digital access divide exerts a statistically significant negative effect on rural residents in eastern China (coefficient equals negative 0.145, p value less than 0.05). This result is consistent with the region’s advanced economic development and high internet penetration, conditions that render connectivity indispensable for schooling, employment, and daily life; exclusion therefore constitutes a barrier to opportunity and, consequently, a threat to psychological well-being. In non-eastern regions—where internet infrastructure is less developed and online services are not yet integral to every domain—the access divide does not significantly influence mental health. Nevertheless, among non-eastern residents who are connected, both the digital use divide and the digital utility divide continue to impede work efficiency and life convenience, producing inferior mental-health outcomes.

Further disaggregation shows that the use and utility divides operate differently across regions. In the east, the estimated coefficient of the digital use divide is negative 0.191 (p value less than 0.001) and that of the digital utility divide is negative 0.105 (p value less than 0.001), indicating that even when infrastructure is available, inability to exploit digital resources and low perceived importance of the internet both depress psychological states. In non-eastern areas the corresponding coefficients are negative 0.116 and negative 0.111 (both p values less than 0.001), demonstrating that these divides are equally harmful. Lower average schooling and slower economic growth in non-eastern regions likely magnify the damage, because residents possess fewer skills with which to convert digital access into tangible benefits.

pages 46:

Panel A of Table 11 examines whether self-assessed social-class perception mediates the effect of the digital divide on the mental health of rural Chinese residents. Column (1) shows that the digital access divide exerts a significant negative effect on mental health (coefficient = −0.110). Yet column (2) indicates that the access divide does not significantly predict self-assessed social class; hence, no mediating role is detected. Turning to the digital use divide, column (4) reports a significant negative coefficient of −0.143 on mental health. Column (5) reveals that the use divide also significantly lowers self-assessed social class. After controlling for the latter in column (6), the coefficient on mental health remains significant and only marginally attenuates to −0.142, implying that the use divide harms mental health partly by depressing perceived social status.

Finally, column (7) documents that the digital utility divide significantly reduces mental health (coefficient = −0.109, p < 0.01). Column (8) shows the same divide significantly lowers self-assessed social class. Introducing the mediator in column (9) leaves the negative effect on mental health virtually intact (coefficient = −0.107), confirming that the utility divide operates, at least partially, through diminished perceptions of social standing.

***Comment7：Clean up formatting issues, especially in Table 1 where items are duplicated. Ensure Figure 1 is clear and labeled.***

Response: Thank you for this suggestion. We have revised Table 1 and Figure 1 accordingly. Duplicate items in Table 1 have been replaced with scored indices to enhance conciseness, and Figure 1 is now embedded in the main text to ensure it is fully accessible and comprehensible to readers.

| Item | | | | Opt | | | Cnt | | N | | Pet | |
| --- | --- | --- | --- | --- | --- | --- | --- | --- | --- | --- | --- | --- |
| Mental Health Levels | | | |  | | |  | |  | |  | |
| How often have you felt pleased in the past week | | | | less than 1 day  1–2 days  3–4 days  5–7 days | | | 868  2900  3435  3911 | | 11114  11114  11114  11114 | | 7.81  26.09  30.91  35.19 | |
| How often have you felt happy in life in the past week | | | | less than 1 day  1–2 days  3–4 days  5–7 days | | | 717  2414  3630  4353 | | 11114  11114  11114  11114 | | 6.45  21.81  32.66  39.17 | |
| How often have you felt down in the past week? | | | | less than 1 day  1–2 days  3–4 days  5–7 days | | | 4378  5092  1021  623 | | 11114  11114  11114  11114 | | 39.39  45.82  9.19  5.61 | |
| How often have you felt that everything is an effort in the past week | | | | less than 1 day  1–2 days  3–4 days  5–7 days | | | 4704  4364  1180  866 | | 11114  11114  11114  11114 | | 42.32  39.27  10.62  7.79 | |
| How often have you had trouble falling asleep in the past week | | | | less than 1 day  1–2 days  3–4 days  5–7 days | | | 5040  3615  1491  968 | | 11114  11114  11114  11114 | | 45.35  32.53  13.42  8.71 | |
| How often have you felt lonely in the past week | | | | less than 1 day  1–2 days  3–4 days  5–7 days | | | 6544  3303  708  559 | | 11114  11114  11114  11114 | | 58.88  29.72  6.37  5.03 | |
| How often have you felt sad in the past week | | | | less than 1 day  1–2 days  3–4 days  5–7 days | | | 5854  4241  627  392 | | 11114  11114  11114  11114 | | 52.67  38.16  5.64  3.53 | |
| How often have you felt sad in the past week | | | | less than 1 day  1–2 days  3–4 days  5–7 days | | | 8681  1797  322  314 | | 11114  11114  11114  11114 | | 78.11  16.17  2.90  2.83 | |
| Digital Divide | | | |  | | |  | |  | |  | |
| Digital Access Divide | | | | NO=1  YES=0 | | | 6826  4288 | | 11114  11114 | | 61.42  38.58 | |
| Digital Use Divide | | | |  | | |  | |  | |  | |
| Do you play online games daily | | | | NO=1  YES=0 | | | 5248  1567 | | 6815  6815 | | 77.01  22.99 | |
| Do you use the internet for learning daily | | | | NO=1  YES=0 | | | 4349  2466 | | 6815  6815 | | 63.82  36.18 | |
| Do you use short videos daily | | | | NO=1  YES=0 | | | 1876  4939 | | 6815  6815 | | 27.53  72.47 | |
| Do you shop online daily | | | | NO=1  YES=0 | | | 3160  3655 | | 6815  6815 | | 46.37  53.63 | |
| Digital Utility Divide | | | |  | | |  | |  | |  | |
| How important is internet learning for work | | | | 1  2  3  4  5 | | | 2350  1273  1873  588  731 | | 6815  6815  6815  6815  6815 | | 34.48  18.68  27.48  8.63  10.73 | |
| How important is the internet for entertainment | | | | 1  2  3  4  5 | | | 1703  1532  2436  737  407 | | 6815  6815  6815  6815  6815 | | 24.99  22.48  35.74  10.81  5.97 | |
| How important is the internet for keeping in touch with family and friends | | | | 1  2  3  4  5 | | | 3858  2458  379  74  46 | | 6815  6815  6815  6815  6815 | | 56.61  36.07  5.56  1.09  0.67 | |
| How important is internet learning for work | | | | 1  2  3  4  5 | | | 2565  1742  1599  478  431 | | 6815  6815  6815  6815  6815 | | 37.64  25.56  23.46  7.01  6.32 | |
| How important is the internet for daily life | | | | 1  2  3  4  5 | | | 2637  1467  1533  611  567 | | 6815  6815  6815  6815  6815 | | 38.69  21.53  22.49  8.97  8.32 | |
| Mediating Variables | | | |  | | |  | |  | |  | |
| class | | | |  | | |  | |  | |  | |
| how would you rate your social status in your local area | | | | 1  2  3  4  5 | | | 865  1583  5030  2069  1567 | | 11114  11114  11114  11114  11114 | | 7.78  14.24  45.26  18.62  14.10 | |
| condition | | | |  | | |  | |  | |  | |
| how would you rate your income level in your local area | | | | 1  2  3  4  5 | | | 1698  1836  4942  1433  1205 | | 11114  11114  11114  11114  11114 | | 15.28  16.52  44.47  12.90  10.84 | |
| fair | | | |  | | |  | |  | |  | |
| how would you rate the wealth gap in our country | | | | 0  1  2  3  4  5  6  7  8  9  10 | | | 224  74  131  366  277  1729  1381  1024  1214  338  1466 | | 11114  11114  11114  11114  11114  11114  11114  11114  11114  11114  11114 | | 2.02  0.67  1.18  3.29  2.49  15.56  12.43  9.22  10.92  3.04  13.19 | |
| Control Variables | | | |  | | |  | |  | |  | |
| Gender | | | | Female=0  Male=1 | | | 5442  5672 | | 11114  11114 | | 48.97  51.03 | |
| edu | | | | Above senior high school=0  Below senior high school=1 | | | 708  10406 | | 11114  11114 | | 6.37  93.63 | |
| marriage | | | | NO=0  YES=1 | | | 1609  9505 | | 11114  11114 | | 14.48  85.52 | |
| religion | | | | NO=0  YES=1 | | | 10824  290 | | 11114  11114 | | 97.39  2.61 | |
| house | | | | NO=0  YES=1 | | | 3953  7161 | | 11114  11114 | | 35.57  64.43 | |
| car | | | | NO=0  YES=1 | | | 8011  3103 | | 11114  11114 | | 72.08  27.92 | |
| [region](https://fanyi.so.com/?src=onebox#region)1 | | | | Non-eastern region=0  Eastern region=1 | | | 6758  4356 | | 11114  11114 | | 60.81  39.19 | |
| [region](https://fanyi.so.com/?src=onebox#region)2 | | | | Non-western region=0  Western region=1 | | | 7304  3810 | | 11114  11114 | | 65.72  34.28 | |
| Item | N | Mean, | Median | | Minimum | Maximum | | StandardDeviation | | Kurtosis | | Skewness |
| age | 11114 | 46.8597 | 48 | | 16 | 89 | | 16.3172 | | 2.0435 | | -0.0569 |
| fml_count | 11114 | 4.3644 | 4 | | 1 | 15 | | 2.0921 | | 4.0849 | | 0.7619 |
| Digital Use Divide | 6815 | -4.43e-17 | 0.0808 | | -1.0962 | 1.1415 | | 0.6576 | | 2.0881 | | 0.1105 |
| Digital Utility Divide | 6815 | -2.95e-17 | -0.0722 | | -1.4784 | 4.1687 | | 0.9797 | | 3.5166 | | 0.6349 |
| Mental Health Levels | 11114 | -1.09e-16 | 0.0967 | | -4.1124 | 1.4671 | | 0.9954 | | 3.2657 | | -0.6532 |

The revised Figure 1 is presented on page 18; it clearly illustrates the foundational research logic of the study.

***Comment8：Edit for grammar and clarity. For example, “55st statistical report” should be “55th statistical report.”***

Response: Thank you for your question. We have revised the issues you raised to ensure the rigor and reliability of the article.

The specific revisions can be found as follows.

In addition to the formatting issues raised by the reviewer, other optimizations for grammar and clarity are also present in the response to Comment 6. For details, see the revisions made in response to Comment 6 above.

***Comment9：Expand on practical implications of the findings and discuss reverse causality more critically.***

Response: Thank you for your comments. We have revised the manuscript as follows: First, we have summarized the key findings more concisely in the text to complement the detailed tables, ensuring that the main results are clearly highlighted and easily accessible. Second, we have expanded the discussion on the practical implications of our findings. The results have significant value for advancing the Digital China strategy and improving public health interventions. We also provide specific policy recommendations for different rural resident groups based on our heterogeneity analysis. Regarding reverse causality, we have critically examined this issue. While acknowledging the potential for reverse causality, we emphasize that our robustness tests (IV estimation, Hausman specification tests, and partial-R² metrics) and extensive robustness checks support the credibility of our core findings. Future research could employ randomized or cross-over designs to further explore these relationships.

The specific revisions can be found on page 51 of the revised manuscript.

Relative to the existing literature, the present study makes two principal contributions. First, we extend the conventional two-tier conceptualisation of the digital divide (access versus use) to a three-tier framework by incorporating the digital utility divide. Prior work has overwhelmingly operationalised the divide as a binary of “access–use”, thereby neglecting the deeper implications of perceived internet utility. Our findings not only furnish robust evidence for the existence of the utility divide, but also dissect its nuanced psychological consequences for rural residents. This theoretical extension furnishes an analytical template for future scholarship. Second, we offer a focused examination of rural populations in a developing-country context, thereby redressing the paucity of mental-health research on this demographic. Rather than restricting attention to canonical “farmers”, we broaden the analytical lens to encompass all agricultural and non-agricultural workers resident in rural areas—an innovation that carries direct implications for place-based policy design. Additionally, by disaggregating the three divides and tracing their differential pathways—via self-assessed social class, self-assessed economic status, and perceived fairness—we provide a level of mechanistic detail that is seldom available in extant studies and that is indispensable for targeted intervention. Notwithstanding these strengths, full resolution of reverse causality remains elusive. Rural Chinese residents confront objectively constrained social channels and cognitive repertoires relative to their urban counterparts. Following Sattar and Øland , two feedback loops are plausible. First, depressive symptomatology may precipitate social withdrawal, eroding the perceived need to go online; low self-efficacy leads individuals to forgo the internet as a means of social or informational expansion, manifesting as access and use divides[64]. Second, depression can impair technology-learning capacity, causing already-connected individuals to abandon skill acquisition and to devalue internet utility—an endogenous process that surfaces as a utility divide. Future work should employ randomised or cross-over designs to neutralise these reciprocal influences[65]. Nevertheless, the battery of endogeneity tests deployed here—IV estimation, Hausman specification tests, and partial-R² metrics—together with an extensive suite of robustness checks, sustains the credibility and policy relevance of the core findings.

***Comment10：Offer more actionable policy recommendations tailored to different demographic groups.***

Response:

Thank you for this helpful suggestion. In the revised Section 7.1 we now translate the heterogeneity results into concrete, age-/gender-/education-/region-specific intervention packages (e.g., mobile-coverage expansion plus short-video training for rural youth; inter-generational video-call tutorials for older villagers; basic-skill courses for low-education groups and advanced-analytics workshops for high-education groups; base-station upgrades in central-western provinces while accelerating gigabit speeds in the east). These actionable measures are presented in running text rather than bullet points and are explicitly linked to the Digital China strategy and grassroots mental-health programmes, providing policymakers with ready-to-implement guidance that goes beyond the usual broad calls for “more infrastructure”.

The specific revisions can be found on page 53 of the revised manuscript.

7Implications

7.1Policy Implications

The results of this study hold significant value for the advancement of the Digital China strategy and public health interventions. The Digital China strategy aims to achieve high penetration and high-quality development of the Internet. The heterogeneity analysis in this study demonstrates the impact of the digital divide on the mental health of different groups of rural residents, which will provide references for the precise advancement of the Digital China strategy. For example, the focus of high penetration development should be shifted to the central and western regions or middle-aged and elderly groups. The focus of high-quality development should be on rural areas in the eastern region or rural residents with higher education levels, thereby reducing the waste of efficiency. As an important part of public health interventions, the response to mental health issues is also of great significance for its optimization. The results show the differences in the mechanisms through which different levels of the digital divide affect the mental health of rural residents, which will provide fundamental intervention pathways for public health interventions to alleviate the mental health problems of digital vulnerable groups such as rural residents, thereby enhancing the effectiveness of interventions.

The results of this study also provide certain value enlightenment for grassroots policy-making. The results show the differences in the impact of the digital access divide, digital use divide and digital utility divide on the mental health of different groups of rural residents, which is conducive to the local governments to propose specific and targeted measures for different groups of rural residents.

Age-Specific Interventions. For rural youth,the strategy should involve expanding mobile internet coverage in remote areas and integrating digital skills training into pre-employment services. This includes incorporating skills like online marketing, remote work software, and short-video creation into training programs, and establishing a digital skills certification system to link skill acquisition with better job opportunities. This approach can enhance digital engagement and mitigate the psychological harm caused by access and use divides. For middle-aged and elderly rural residents, emphasizing the internet’s role in maintaining intergenerational connections and reducing caregiving burdens can alleviate their resistance to digital technology and the psychological impact of the use divide.

Gender-Specific Interventions.For women, efforts should focus on breaking down barriers to internet access and use, providing sustained digital tool training to reduce the psychological impact of these divides. For men, the emphasis should be on addressing the adverse effects of potential internet risks, enhancing their recognition of the internet’s value and safety, and thereby mitigating the negative impact of the digital utility divide.

Education-Specific Interventions.For rural residents with lower education levels, promoting basic digital skills training can help overcome cognitive barriers and reduce the psychological impact of the digital divide. For those with higher education, advanced digital skills training, such as digital marketing analysis, should be promoted to create more high-quality job opportunities and prevent skill-demand mismatches.

Region-Specific Interventions.In eastern rural areas, improving internet speed and stability while increasing the frequency of digital applications in daily life is essential. In central and western rural regions, priority should be given to ensuring basic internet access and optimizing base station construction and signal strength, along with long-term plans for digital skills enhancement. These measures can effectively reduce the adverse effects of the digital divide.

We have made every effort to revise the manuscript. In the revised version, changes are highlighted in red without compromising the content and structure of the paper. Moreover, we have had the manuscript polished by a professional editor to enhance the language quality. The revised version is now significantly improved. We sincerely thank the Editor/Reviewers for their hard work and suggestions and hope that the revisions will be approved. Best regards!
